# Nocardiosis in a Kidney-Pancreas Transplant

**DOI:** 10.1155/2010/573234

**Published:** 2010-01-26

**Authors:** I. Fontana, G. Gasloli, A. Magoni Rossi, C. Bornacina, F. Dodi, M. Bertocchi, O. Soro, P. Diviacco, A. De Negri, E. Bocci, C. Ferrari, A. Giannone, Umberto Valente

**Affiliations:** ^1^Department of Transplantation, San Martino University Hospital, L.go R. Benzi 10, 16132 Genoa, Italy; ^2^Department of Dermatology, University of Western Pindemont, via Solaroli 17, 28100 Novara, Italy

## Abstract

34-year-old man with chronic renal and pancreas failure in complicated diabetic disease received a kidney-pancreas transplantation. On the 32nd postoperative day, an acute kidney rejection occurred and resolved with OKT3 therapy. The patient also presented refractory urinary infection by *E. Fecalis* and *M. Morganii*, and a focal bronchopneumonia in the right-basal lobe resolved with elective chemotherapy. During the 50th post-operative day, an intense soft tissue inflammation localized in the first left metatarsal-phalangeal articulation occurred (Figure 1) followed by an abscess with a cutaneous fistula and extension to the almost totality of foot area. The radiological exam revealed a small osteo-lacunar image localized in the proximal phalanx head of the first finger foot. From the cultural examination of the purulent material, *N. Asteroides* was identified. An amoxicillin-based treatment was started and continued for three months, with the complete resolution of infection This case is reported for its rarity in our casuistry, and for its difficult differential diagnosis with other potentially serious infections.

## 1. Introduction

*Nocardia* are weakly gram-positive, filamentous bacteria found worldwide in soils [1], members of the family Nocardiaceae, the aerobic actinomycetes. *Nocardia Asteroides* is the principal cause of systemic nocardiosis in the United States [2]. 

Immunosuppression is the main risk factor for nocardical infections as well as the majority of nocardical infections occurs in severely immunocompromised patients (with decreased cellular-mediated immunity). The frequency of nocardical infections in solid organ transplant recipients varies between 0.7% and 3% and has mostly been reported in heart, kidney, and liver transplant recipients, and less frequently in lung transplantation [3]. 

The infection is usually acquired via inhalation of the microorganisms, which allows the establishment of a focal pneumonitis in 75% of cases and, in the half of these, hematogenous dissemination, or oligo-symptomatic nonapparent manifestations. Forms with cerebral abscess occur in 25%. However, cutaneous dissemination, which occurs in 10%, is most commonly presented as cutaneous abscess. In this cases a cutaneous dissemination is a manifestation of an opportunistic severe disease. Nocardiosis can be an acute, subacute, or chronic suppurative infection. 

The 90% of nocardical pneumonias are caused by *Nocardia Asteroides* complex [2]. Pulmonary nocardiosis can have many responses ranging from granulomatous to purulent reactions [1–4]. Patients with pulmonary nocardiosis typically present with dispnea, cough, or pleuritic chest pain in addition to fever, malaise, and anorexia [5]. Radiological examination usually demonstrates irregular nodular lesions, which may progress to cavitation. They may also appear as diffuse pneumonic infiltrates or consolidative with pleural effusions [6]. As the diagnosis of pleurical nocardiosis is done, it should be assumed that the immunosuppressed patient has a disseminated disease. The differential diagnosis of a syndrome with pulmonary and brain nodular lesions should include *Nocardia* as well as *Asperegillus* spp., mycobacteria, *Rhodococcus equi,* and *Crypotococcus Neoformans *[7]. There has been no effective measure to prevent inhalation; however, it seems that trimethoprim-sulfametoxazole prophylaxis (used for *Pneumocysis Jiroveci* infection in the first six months of transplantation) may actually reduce the incidence of disease [2]. 

Penetrating cutaneous injury can be, although rarely, an inoculation way. Cutaneous nocardiosis can present as an acute superficial skin infection with pustules, abscesses, pyoderma, and cellulitis or as a lymphocutaneous infection [1–8]. The definitive diagnosis of nocardiosis requires a demonstration of the organism on a culture from a suspected site.

## 2. Case Report

A 34-year-old man with chronic renal and pancreas failure in complicated diabetic disease received a kidney-pancreas transplantation. The perioperative prophylaxis was ampicillin and cefoxatime; immunosuppressive therapy was made with steroids, antilymphocyte globulin (ALG), and Cyclosporine. On the 32nd post-operative day an acute kidney rejection occurred and resolved with anti-CD3 monoclonal antibody (OKT3) therapy. The patient also presented refractary urinary infection by *Enterococcus Fecalis* and *Mycobacterium Morganii*, treated with elective chemiotherapy with amoxicillin and ciprofloxacin, and a focal bronchopneumonia in the right-basal lobe. 

**Figure 1:**
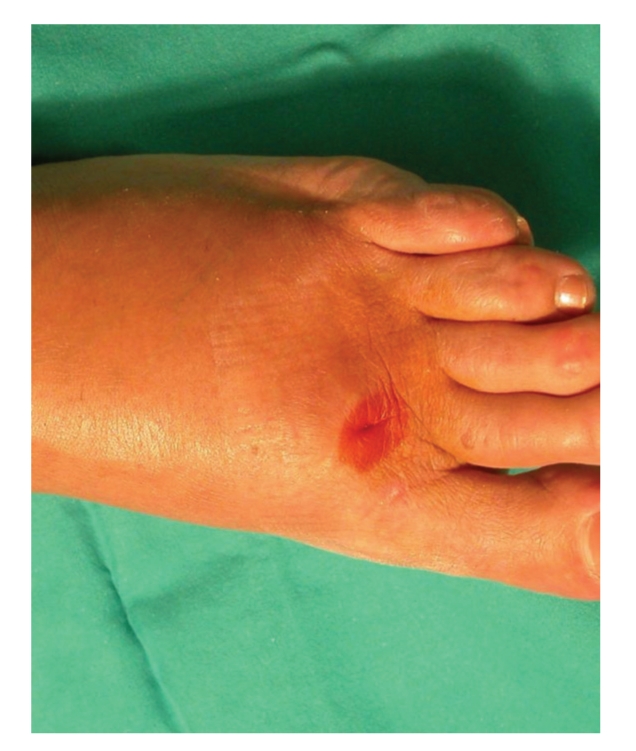
Nocardiosis: soft tissue inflammation localized in the left metatarsus-phalangeal articulation, associated with a cutaneous fistula, occurred during the 50th post-operative day.

During the 50th post-operative day, an intense soft tissue inflammation localized in the first left metatarsus-phalangeal articulation (Figure 1) followed by an abscess with a cutaneous fistula and extension to the almost totality of the foot area was found. The standard radiological exam revealed a small osteo-lacunar image localized in the proximal phalanx head of the first finger foot. From the cultural examination of the purulent material, *Nocardia Asteroides* was identified. An amoxicillin-based treatment was immediately started and continued for three months, with the complete resolution of infection.

## 3. Discussion

This case is reported for its rarity in our casuistry, starting from 1985, for its difficult differential diagnosis with other potentially serious infections in immunosuppressed patients (tuberculosis, nontubercular mycobacteriosis, mycosis, other bacterial infections) or rheumatic pathology. The documentation of the infection presupposes noncutaneous dissemination of infection. 

In our case report, the elective chemotherapy, established after a definitive and early diagnosis, based on the demonstration of the microorganism on a culture from the purulent material, resulted in the complete resolution of infection.
